# PRFS-Based MR Thermometry Versus an Alternative T1 Magnitude Method – Comparative Performance Predicting Thermally Induced Necrosis in Hepatic Tumor Ablation

**DOI:** 10.1371/journal.pone.0078559

**Published:** 2013-10-24

**Authors:** Christian Rosenberg, Antje Kickhefel, Birger Mensel, Tilman Pickartz, Ralf Puls, Joerg Roland, Norbert Hosten

**Affiliations:** 1 Institute of Diagnostic Radiology and Neuroradiology, University Medicine Greifswald, Greifswald, Mecklenburg-Vorpommern, Germany; 2 Clinic of Internal Medicine, Division of Gastroenterology, Endocrinology and Nutritive Medicine, University Medicine Greifswald, Greifswald, Mecklenburg-Vorpommern, Germany; 3 Siemens Healthcare, Erlangen, Bavaria, Germany; The Chinese University of Hong Kong, Hong Kong

## Abstract

**Objective:**

To compare the accuracy of a semi-quantitative proton resonance frequency shift (PRFS) thermal mapping interface and an alternative qualitative T1 thermometry model in predicting tissue necrosis in an established routine setting of MRI-guided laser ablation in the human liver.

**Materials and Methods:**

34 cases of PRFS-guided (GRE) laser ablation were retrospectively matched with 34 cases from an earlier patient population of 73 individuals being monitored through T1 magnitude image evaluation (FLASH 2D). The model-specific real-time estimation of necrotizing thermal impact (above 54 °C zone and T1 signal loss, respectively) was correlated in size with the resulting necrosis as shown by lack of enhancement on the first-day contrast exam (T1). Matched groups were compared using the Mann-Whitney test.

**Results:**

Online PRFS guidance was available in 33 of 34 cases. Positive size correlation between calculated impact zone and contrast defect at first day was evident in both groups (p < 0.0004). The predictive error estimating necrosis was median 21 % (range 1 % - 52 %) in the PRFS group and 61 % (range 22 - 84 %) in the T1 magnitude group. Differences in estimating lethal impact were significant (p = 0.004), whereas the real extent of therapy-induced necrosis showed no significant difference (p > 0.28) between the two groups.

**Conclusion:**

PRFS thermometry is feasible in a clinical setting of thermal hepatic tumor ablation. As an interference-free MR-tool for online therapy monitoring its accuracy to predict tissue necrosis is superior to a competing model of thermally induced alteration of the T1 magnitude signal.

## Introduction

The ability to acquire near-real-time maps of in vivo body and tissue temperature superiorly makes magnetic resonance imaging (MRI) a well-suited modality for guiding and monitoring minimally invasive thermal therapies [1-6].

Ablative hyperthermal tumor treatment is based on high-temperature regimens applying temperatures of 50 - 80 °C or more. The aim is to induce tissue coagulation and acute necrosis through processes such as protein coagulation. To achieve this, the entire target tumor volume must be exposed to an adequate temperature for a certain period of time [7,8]. At the same time destruction of healthy surrounding tissue needs to be minimized and harm to sensitive neighboring structures avoided. Heat distribution depends on the type of energy applied – laser, radiofrequency, microwave or noninvasive high-focused ultrasound (HIFU) – as well as the structure and architecture of the target tissue [9,10]. Thermal conductivity of the target tissue may alter during therapy when inducing protein denaturation. Neighboring vasculature may diminish heat-induced therapy effects through mechanisms such as perfusion and diffusion. Effective heat distribution is partly non-predictable and needs online monitoring. As with other local cancer treatments including surgery, monitoring of success at the time of treatment requires independent parameters to define a treatment end point and to identify therapy-related adverse events [11].

Success of MRI temperature monitoring depends on the accuracy of temperature estimation. Out of a variety of different approaches, including proton density, T1 and T2 relaxation times, diffusion coefficient, and magnetization transfer, the proton resonance frequency shift (PRFS) method is the only one that offers a linear relationship to temperature and low susceptibility to tissue quality [12-21]. All MR thermometry models in common are in a state of quality assessment and rely on animal or ex vivo models. Data on the use of PRFS-monitored laser ablation in humans have been obtained for soft tissue tumors of the neck region and intradiscal laser ablation in the spine [13,22]. Even though the method is known for more than 10 years there is only few data in the literature on using the PRFS model both in moving organs and in a clinical routine approach. Out of these, two groups reported the use of PRFS phase mapping to monitor therapy effects of radiofrequency-induced thermal ablation in the human liver [23-25]. Lacking sufficient filters to deal with radiofrequency interference, ablation and MR data acquisition are often interleaved and therefore do not meet requirements of online monitoring in this specific setting. Laser ablation, unlike all other current modalities, is an interference-free MRI-guided ablation technique and hypothetically well suited to be monitored through PRFS thermal mapping. In a recent study an adequate congruence between calculated lethal dose and resulting necrosis in hepatic tumor ablation was reported [26]. Apart from image postprocessing the same model still lacks a proof of operability for the virtual online interface, which the interventional radiologist relies on. At the same time comparative studies on different model’s performances are underrepresented in the literature.

The objective of this study was to compare the accuracy of a semi-quantitative PRFS thermal mapping interface [25,26] and an alternative qualitative T1 thermometry model in predicting tissue necrosis in an established routine setting of MRI-guided laser ablation in the human liver. 

## Materials and Methods

### Patients and pair matching

Study approval from the institutional ethics committee (registration number BB 93/08, Ethics Committee of the Ernst Moritz Arndt University Greifswald) was obtained. Thirty-four PRFS-guided percutaneous ablative procedures for primary and secondary malignancies of the liver were evaluated. All 18 patients hosting the target tumors gave written informed consent and were included in the study protocol. Individuals were chronologically recruited from the group of patients being scheduled (2008-2009) for local ablative therapy in our institution. An institutional interdisciplinary tumor board stated indications applying guideline-based oncologic criteria [27]. Individual therapies comprised single procedures for solitary metastases in 16 cases, multiple procedures for initial disease in 10 cases, and independently, representation for recurrent hepatic metastasis in 4 cases and reablation of locally recurrent tumor in 4 cases. The last were considered solitary tumors as far as technical data analysis was concerned. 

Cases were retrospectively matched with cases of hepatic MR-guided tumor ablation from an older series (2004-2008) comprising 73 patients (table 1). Thermal monitoring in these cases was accomplished through repetitive acquisition of T1 magnitude images. The resulting matched-pair cohort consisted of 68 cases. Cases were matched for tumor size (maximum diameter) in first place and tumor localization (liver segment) in second place; entity of primary tumor, patient sex and age, in the order of appearance, were subordinated criteria (tables 1+S1).

**Table 1:** Comparison of matched-pair groups, PRFS-guided vs. T1-magnitude-guided ablative procedures.

**MRI thermometry**	**PRFS**	**T1 magnitude**
**Patients (n)**	18 (13 male, 5 female)	22 (14 male, 8 female)
**Procedures (n)**	34	34
**Primary tumor type (n)**	8	7
Colorectal carcinoma	24	20
Hepatocellular carcinoma	2	3
Cholangiocellular carcinoma	1	1
Pancreatic carcinoma	1	2
Breast carcinoma	1	6
Endometrium carcinoma	3	-
Malignant melanoma	1	-
Gastric carcinoma	1	-
Nasopharyngeal carcinoma	-	1
Neuroendocrine carcinoma	-	1
**Age (yrs)**	64.8 (52-80)	64.4 (37-83)
**Maximum target diameter (cm)**	2.3 (0.5-5.6)	2.0 (0.7-6.0)
**Liver segment (n)**		
S2	5	1
S3	1	2
S2/3	-	2
S4	4	1
S4/8	1	1
S5	4	-
S5/6	1	1
S6	9	4
S6/7	1	2
S6/7/8	1	-
S7	2	10
S7/8	-	1
S8	5	8
S8/5	-	1
**Applicators (n)**	2 (1-4)	2 (1-4)
**Applied energy (kJ)**	36.4 (12.2-65.2)	34.0 (15.5-69.0)

### Ablation Procedure

Procedures were identical in both groups. They were fully performed in the MRI suite using a closed 1.5 T MR scanner with flexible spine/body array coils and in-room console (MAGNETOM Avanto for the current series, MAGNETOM Symphony for the earlier series; both Siemens AG, Erlangen, Germany) according to an established protocol [26,28]. The same experienced (7 years) interventional radiologist performed all 68 interventions. 

Percutaneous laser ablation was performed using a miniaturized internally cooled applicator system (RoweCath®; RoweMed, Parchim, Germany). It consisted of a 5.5-French polytetrafluoroethylene tube carrying a titanium mandrin for catheter placement. The mandrin was later replaced by an optical laser fiber with a flexible diffusor tip of 3 cm length. Three separate Nd:YAG laser sources (Medilas fibertom; Dornier, Wessling, Germany) operating at a wavelength of 1064 nm were fitted with optional two- and four-time beam splitters providing a variety of setting designs for simultaneous use of multiple fibers [28,29].

Procedural planning and guidance were performed on the basis of fast axial T1-weighted gradient-recalled echo (GRE) sequences (3-dimensional fast low-angle shot (FLASH 3D) or volume-interpolated breath-hold examination (VIBE)) in breath-hold technique [28]. Ventral insertions were performed under sterile conditions through a convenient opening of the body array coil. Initial insertion and optionally repositioning of the applicator were performed outside the magnet interleaved with image acquisition for position control.

Hepatic tumors were treated using single or multiple applicators simultaneously. In general, tumors larger than 2 cm in diameter were treated with at least two applicators in parallel position. When a single applicator was used, it was positioned in the middle of the tumor, piercing the two opposite margins. When multiple applicators were used, overlapping (at least 5 mm were mandatory) ellipsoid impact zones with the length of the active tip and a maximum width of 2.5 cm were estimated. Continuous laser application was performed according to a standard regimen, the wattage was increased in increments of 2 W/min, and the maximum energy of 14 W was maintained for another 17 min. Definite therapy success in all 68 procedures was determined in the 24-hour dynamic contrast-enhanced MRI study with a target being fully covered by the contrasting defect at portal venous phase.

### PRFS-based thermal monitoring

**Figure 1:**
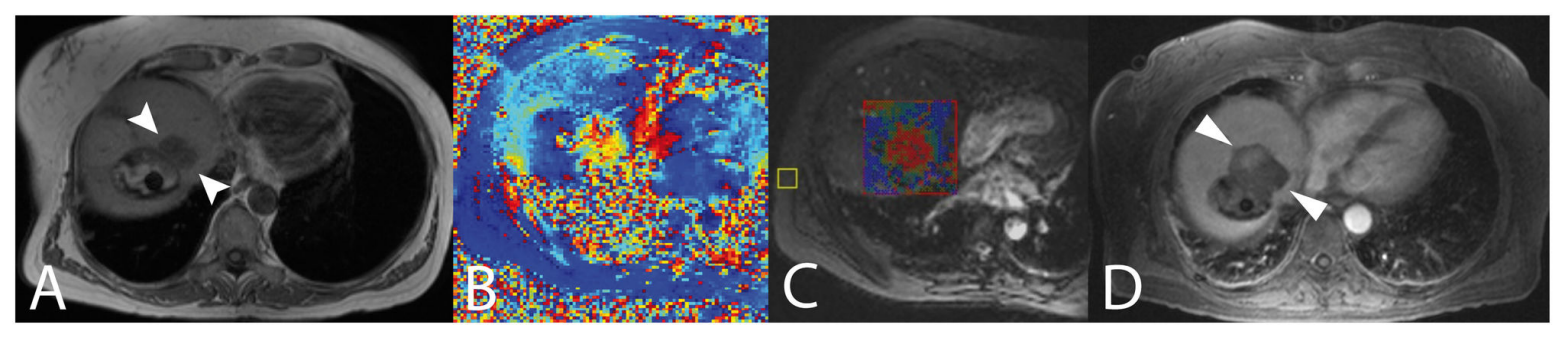
MR-guided therapy control. Example case (A) of an initially T1-hypointense recurrent tumor (arrowheads) at the margin of an older inhomogeneously hyperintense ablation zone. Phase difference image derived from subtracting a non-heating reference image (B) and thermal map with color-coded pixels in a quadrate ROI at peak temperature as being displayed on screen during the procedure (C), fast GRE sequences at 1.5 T Magnetom Avanto, Siemens Healthcare, Erlangen, Germany; TE 12 ms, TR 970 ms, BW 260 Hz/pixel, flip angle 65°, field of view (FOV) 320 mm, matrix size 128x128, slice thickness of 3 mm, fat suppression. Color-codes of phase image and online ROI are unequal. The last picture (D) shows the ablation-induced necrosis (arrowheads) demarcated as a lack of extracellular contrast uptake at portal venous phase on 24 h CEMR.

**Figure 2:**
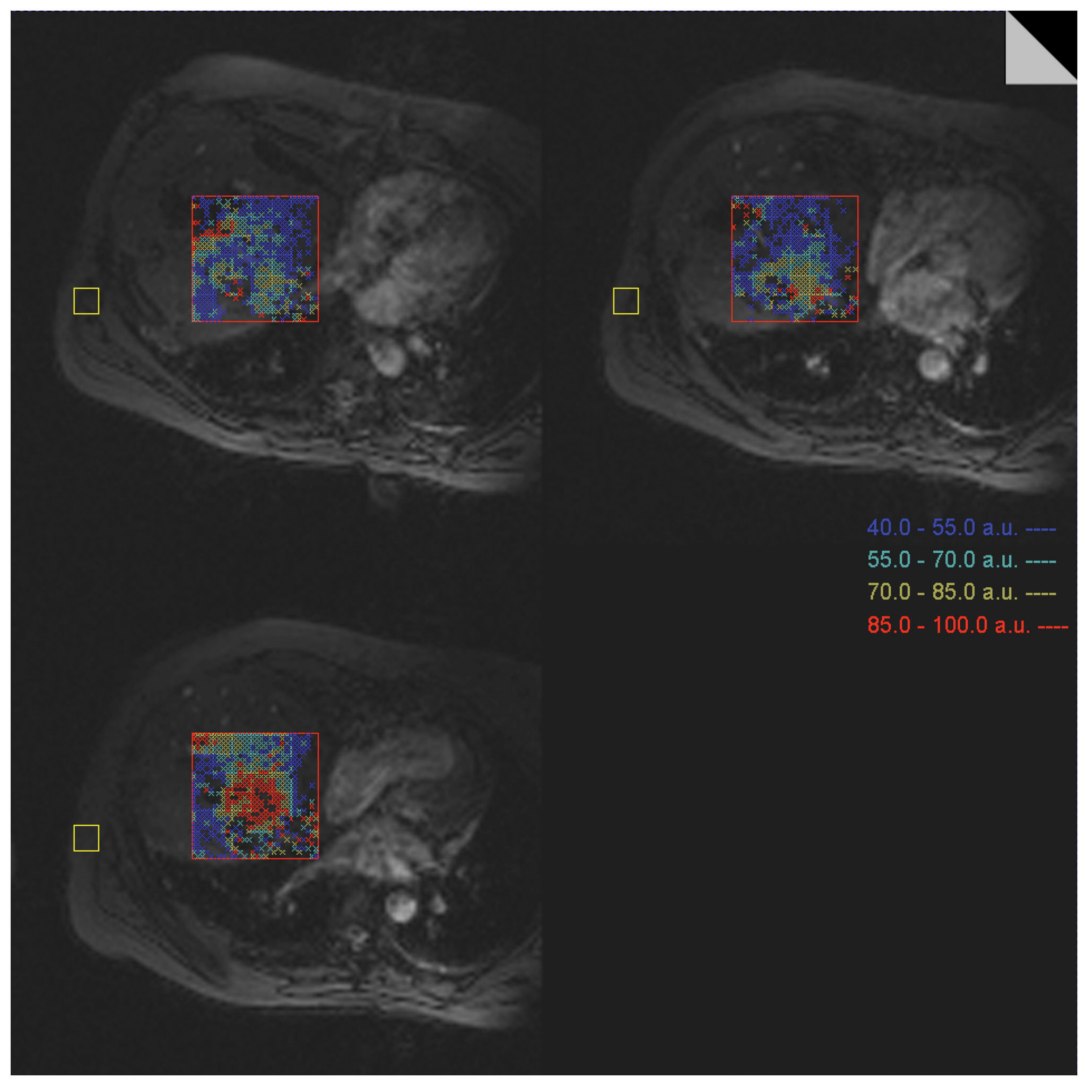
Surveillance screen for online thermal monitoring during the ablation procedure. Repetitive acquisition (1 per breathing cycle) of three parallel slices (fast GRE sequences at 1.5 T Magnetom Avanto; TE 12 ms, TR 970 ms, BW 260 Hz/pixel, flip angle 65°, field of view (FOV) 320 mm, matrix size 128x128, slice thickness of 3 mm, fat suppression). The patient constantly remains in the magnet. Color-coded pixels retrieved from the actual phase difference image display consecutive isothermal zones within a ROI of preselected size and position (red square). For correction of the magnetic field drift (B_0_ correction) a significantly smaller ROI (yellow square) was set into a motion-free area of the magnitude image. SNR of the magnitude image was median 10 ± 2 for all patients investigated. The standard deviation of temperature was median 6 ± 4 °C in non-heated liver.

Once laser fibers were properly positioned in the target zone, the table and patient remained within the magnet. Continuous thermometric imaging was achieved through repetitive acquisition of T1-weighted fast GRE sequences at 1.5 T (Magnetom Avanto, Siemens Healthcare, Erlangen, Germany; TE 12 ms, TR 970 ms, BW 260 Hz/pixel, flip angle 65°, field of view (FOV) 320 mm, matrix size 128x128, slice thickness of 3 mm, fat suppression), as already described and validated [28]. Based on temperature-dependent changes of the proton chemical shift temperatures could be calculated from the phase difference between actual phase (heated) and reference phase (non-heated) (Figure 1). For correction of the magnetic field drift (B_0_ correction) a small ROI (36 voxels) was set into a motion-free area of the magnitude image, distant to the impact zone and providing a signal of maximum intensity and homogeneity, conveniently the autochthonous back muscles. Image acquisition was breath-triggered using a respiratory bellows. Three parallel slices, magnitude and phase image each, with a gap of 6 mm were acquired within one breathing cycle and preferably through the plane defined by two parallel laser fibers or any otherwise expected maximum heat extension (Figure 2). The examiner initially defined sequence repetition counts to last for the time of procedure. Color-coded pixels virtually displayed isothermal zones of homogeneously defined four temperature corridors (dark blue 40-54 °C, light blue 55-69 °C, yellow 70-85 °C, red 85-100 °C) within a quadrate ROI of preselected size (approximately 10 or 15 cm^2^, 400 or 900 voxels) and position on the T1 magnitude image, which was displayed in a separate surveillance window (standard temperature display, Syngo®; Siemens, Erlangen, Germany).

### T1 magnitude thermal monitoring

In the 34 matched cases of T1 magnitude thermal monitoring, heat distribution was visualized through repetitive acquisition of T1 FLASH 2D sequences (TE 4,8 ms, TR 100 ms, BW 260 Hz/pixel, flip angle 70°, slice thickness 5 mm, fat saturation). Ten to fifteen axial slices per acquisition were sufficient to cover the target region. A rise in tissue temperature increased the T1 relaxation time, resulting in a lower T1 signal [30]. Only a signal drop of approximately 25 % was sufficiently visualized through blackening of the regular T1 grey scale. Lethal temperatures of above 60 °C were presumed within the zone of signal loss in the magnitude image according to Puls et al. [31]. Derived from earlier practice the margin of the signal loss qualitatively determined lethal impact and anticipated tissue necrosis [31,32].

### Data analysis

A postprocessing temperature fit and semi-automated segmentation algorithm for the 34 PRFS procedures was described elsewhere and used as internal control in the actual study [26]. With a given standard therapy regimen and continuous mapping of the temperature course a procedure-specific lethal temperature threshold for the peak temperature time point can be calculated according to thermal damage models. Both the Arrhenius damage integral and the peak temperature model revealed a threshold of 52 °C for irreversible cell damage in the actual therapy set-up. Segmentation and registration (MatLab 6.0 Mathworks; Natick, MA) of lethal zones on the postprocessed phase image and evident necrosis according to the perfusion defect in the first day control revealed an 87.2 % conformity at temperatures above 52 °C [26]. As a result a lethal temperature threshold of 55 °C at peak temperature was assumed for daily practice in the given therapy procedure. Based on these results the threshold, had to be set to 55 °C in the actual study. The threshold value represents a semi-quantitative determination of the lethal impact zone and is discriminated in the online ROI, visible to the interventionalist. 

For both groups the ensuing ablative necrosis was determined in the contrast-enhanced first-day control exam (PRFS group: TR 115 ms, TE 5 ms, 30 slices, flip angle 70°, same voxel size and orientation as GRE thermal map; T1 group: TR 100 ms, TE 4.79 ms, 9 slices, flip angle 70°, slice thickness 5mm, axial). Therapy-related tissue necrosis was defined as non-enhancing liver on portal phase control MR scans (Gadovist; Bayer-Schering, Berlin, Germany). For the PRFS group the intraprocedural thermal map at peak temperature within the region of interest (ROI) was correlated with the corresponding axial or angled axial plane in the following manor (Figure 1). Peak temperature was presumed for the last triplet of images acquired during a standardized procedure that lasted 20 min. Out of the three parallel slices the largest zone of thermal impact was chosen. For the T1 group the slice carrying the largest thermal impact zone was chosen from the axial slice package at peak temperature. Planes in both groups were manually segmented and measured in square millimeters (Syngo®; Siemens, Erlangen, Germany). 2D analysis of thermal maps (maximum extent of above 54 °C zone or T1 signal loss, respectively) and corresponding necrosis was performed in consensus by two experienced radiologists, with 7 and 15 years of experience. The Spearman's rank correlation coefficient was calculated for the resulting two data columns in both groups (significance level 0.01). Matched groups were compared using the Mann-Whitney test at a significance level of 0.05.

To assess image quality two experienced (7 and 15 years) observers independently assessed image quality on the thermal maps at peak temperature and arrived to an agreement in later consensus reading. Conspicuities of target tumors and liver morphology as well as delineation and completeness of the thermal impact zone on the discriminative slice were scored on a three-point scale (insufficient, acceptable, or sufficient) and conspicuity of fiber artifact was evaluated (present or not present). 

In the PRFS dataset the signal-to-noise ratio (SNR) was determined for the magnitude image in non-heated liver parenchyma, using a quadrate ROI of 6x6 voxels, dual acquisition and image subtraction (SNR = √2 x S_1_/SD_1-2_) [33,34]. S1 represents the mean signal intensity in non-heated liver from the reference magnitude image as displayed in the ROI, whereas SD_1-2_ is the standard deviation within the same ROI on an image resulting from subtracting the reference from the monitoring image of interest. The SNR was averaged over the three slices for each acquisition. To estimate motion artifacts the temperature SD was determined for the same time points and ROI. 

## Results

### Feasibility and imaging quality of PRFS-based thermal mapping

Real-time thermal monitoring through application of the respiratory-triggered GRE sequence was feasible in all but one of the 34 laser ablation procedures. In one case losing the path to the primary reference image data during the intervention led to dysfunction of the thermal map display - only the background magnitude image could be followed during intervention. This single case was excluded from further analysis.

The SNR of the magnitude images, averaged over three slices of the peak temperature triplet each, ranged from 5.1 to 14.1 (median 10 ± 2) for all patients investigated. The standard deviation of temperature was median 6 ± 4 °C (range 0.9 to 10.2 °C) as being measured on the phase difference image in non-heated liver. 

### Comparative imaging quality of thermal mapping

**Figure 3:**
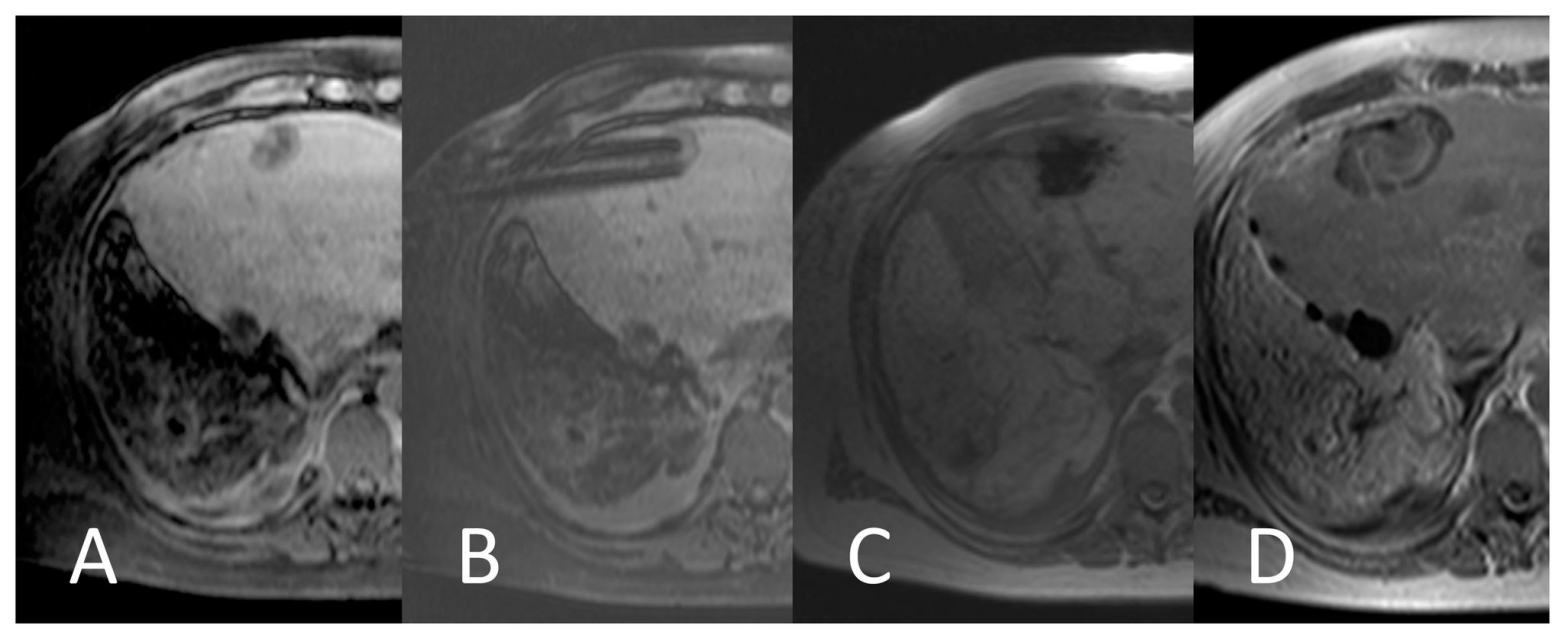
T1 magnitude thermal imaging in a dual applicator ablation of hepatic colorectal carcinoma metastasis. Preablation T1 (A), mandrin placement (B), peak temperature with T1 signal loss (T1 FLASH 2D: TE 4,8 ms, TR 100 ms, BW 260 Hz/pixel, flip angle 70°, slice thickness 5 mm, fat saturation) (C), necrotic contrast defect on 24 h CEMR (D).

**Figure 4:**
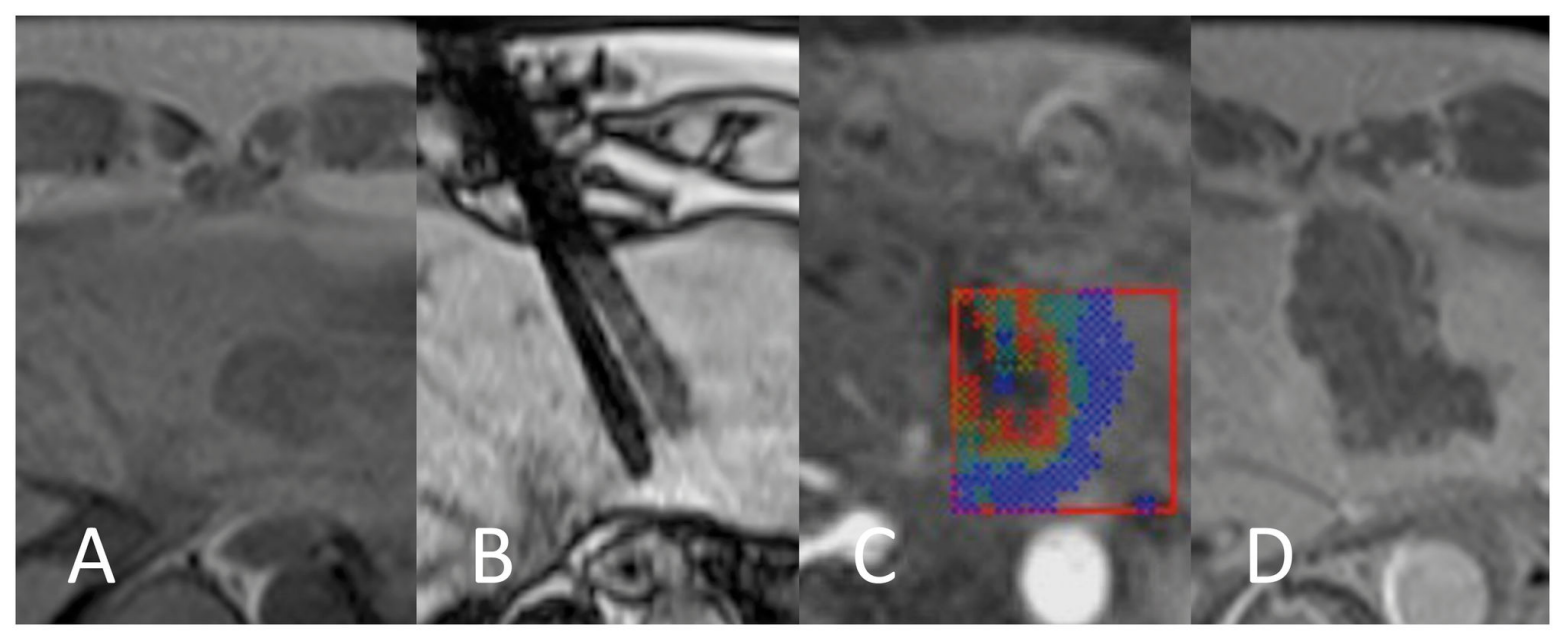
PRFS thermal mapping in a case of dual applicator ablation of hepatic breast cancer metastasis. Preablation T1 (A), mandrin placement (B), peak temperature with 55 °C isothermal line – dark blue/light blue (C), necrotic contrast defect on 24 h CEMR (D).

Qualitative evaluation comprised assessment of anatomic background visualization on the underlying T1 magnitude image in both groups as well as reading of the thermal map for lethal temperature extension. Results are shown in table 2. Conspicuities of target tumor and liver morphology were found acceptable in both modalities (Figure 3 + Figure 4). PRFS thermometry was found significantly advantageous when it comes to delineation of the impact zone. 

**Table 2:** Comparative image quality in PRFS and T1 magnitude thermometry.

MRI thermometry	PRFS(%)	T1 magnitude(%)
Conspicuity of target tumor		
acceptable	54.5	58.8
sufficient	45.5	41.2
Conspicuity of liver morphology		
acceptable	60.6	61.8
sufficient	39.4	38.2
Delineation of thermal impact zone		
acceptable	15.2	35.3
sufficient	84.8	64.7
Completeness of thermal impact zone		
acceptable	30.3	44.1
sufficient	69.7	55.9
Conspicuity of laser fibres		
yes	12.1	35.3
no	87.9	64.7

For the vast majority of PRFS cases the laser fiber neither was displayed nor led to any disturbing artifact on magnitude or phase images. In individual sequences (n = 4) a preheating artifact diameter to the maximum of 1.9 mm was measured. Fibers never showed any pixel correlate in the PRFS thermal map itself, which displayed homogeneous heat distribution in the center of the target zone. Influence of tumor localization within the liver, entity of primary tumor, and count of applicators on feasibility and quality of real-time thermal monitoring were found not to be statistically significant in the patients investigated.

### Outcome and comparative accuracy in the matched-pair analysis

Ablative treatment of target tumors was technically successful in all 34 PRFS-guided procedures as stated in consensus of two radiologists and on the basis of the routine first-day control exam. Four hepatic tumors were electively treated in more than one session comprising calculated partial ablation in the first therapy session, all of these due to tumor size. In one patient with solitary gastric carcinoma metastasis, residual tumor after three consecutive treatment sessions was accepted, as the primary goal of treatment was cytoreduction. In the remaining 17 patients ablative therapy led to elimination of known tumor burden. No therapy-related deaths or major complications occurred. Minor complications comprised occasional peri- or postprocedural pain and self-limited small subcapsular hematoma in one case. During therapy no adverse events or unexpected organic limitations occurred that could have influenced the course of the ablation regimen with or without appearance on the monitor image. 

In all 33 of 34 PRFS cases displayed thermal maps intraprocedurally were read as “therapy goal accomplished”, meaning full coverage of target tumor tissue, consecutive partial ablations in 4 tumors or tolerated residual tumor in one patient. Immediate bedside readings were qualitatively found approved in the 24 h follow-up imaging.

**Figure 5:**
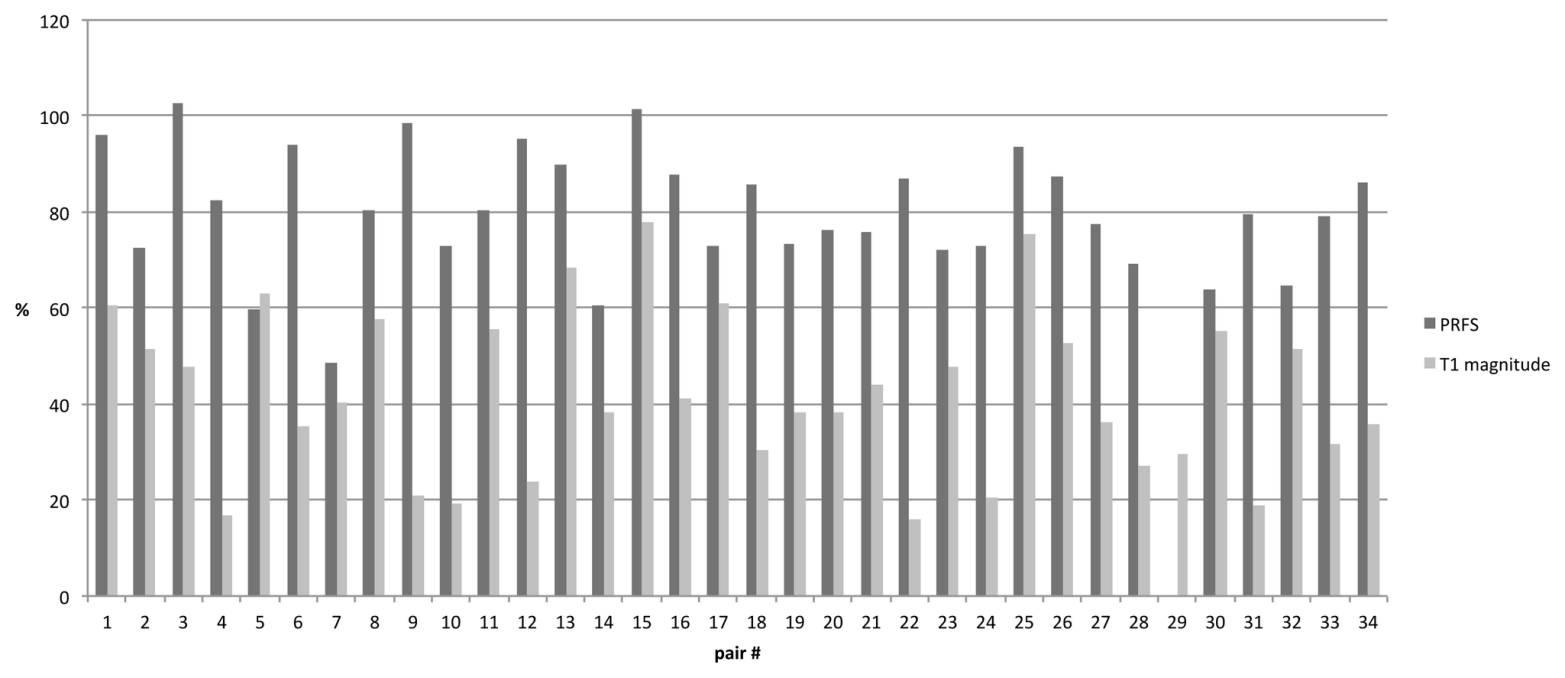
Matched-pair analysis of PRFS and T1 magnitude thermal monitoring. Comparative display of 2D size correlation (cm^2^) between above 54 °C isothermal zone and necrosis in PRFS group (dark column) on the one hand and T1 signal loss and necrosis in the T1 magnitude group (light column) on the other hand. The estimated necrosis (thermometry) is delineated in parts per hundred, with the resulting necrosis (24h CEMR) representing 100 %. Positive correlation of estimated and ensued impact zones was statistically significant for both groups (p < 0.0004). Overestimation (average 2.2 % in 2 cases) only was found when using PRFS thermometry; peak correlation with the approved necrosis in the T1 magnitude group was 78 %.

**Figure 6:**
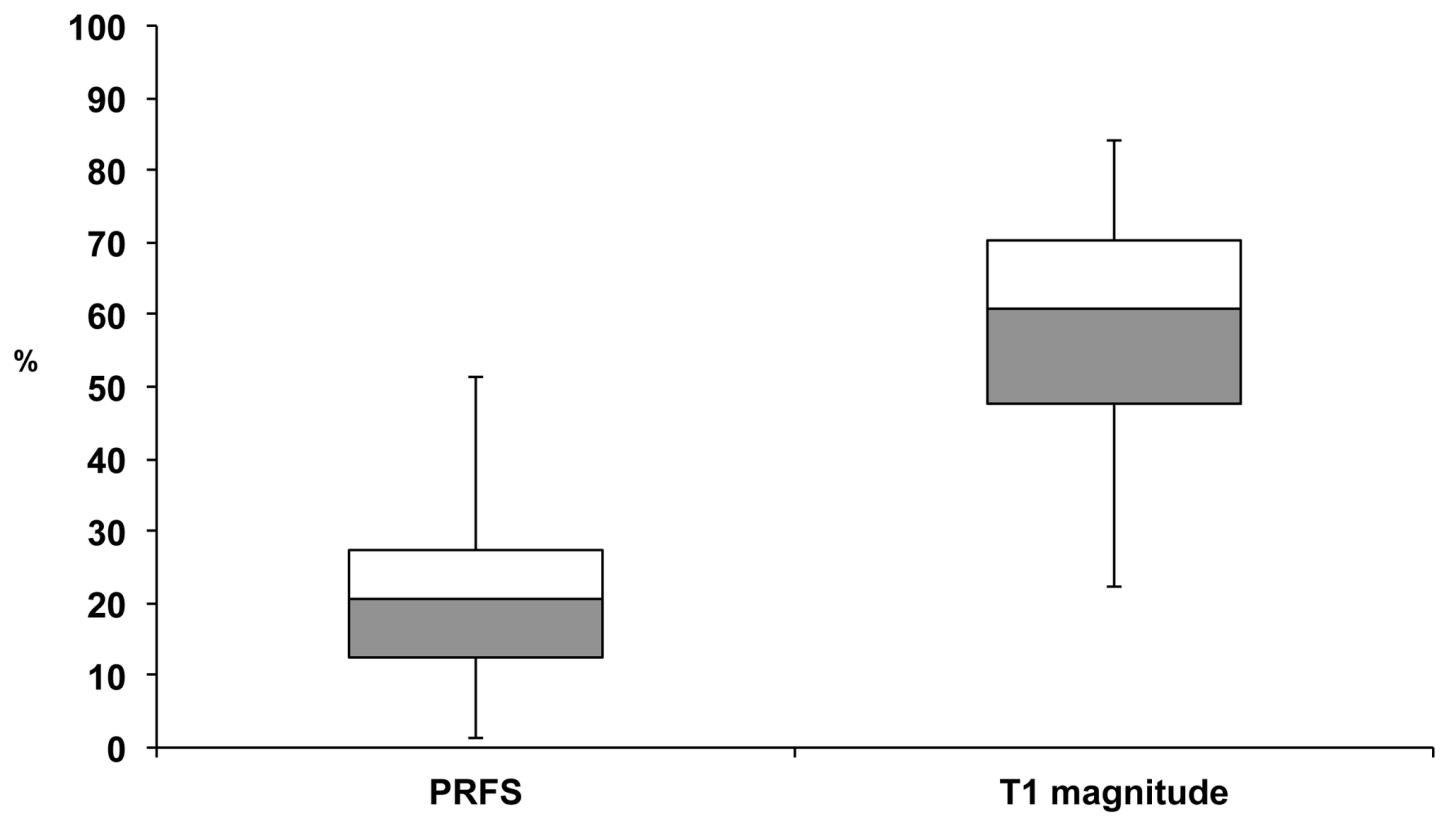
Box blot analysis of predictive errors for PRFS and T1 magnitude thermal monitoring. Values (parts per hundred) of over- and underestimation were evaluated in the PRFS group (range 1 % - 52 %), of underestimation alone in the T1 magnitude group (range 22 % - 84 %). The predictive error is almost threefold the amount with the older T1 magnitude method (median 61 % as compared with 21 %).

Retrospective semi-quantitative 2D size analysis of both the visualized PRFS thermal zone of above 54 °C and the enhancement defect at first-day control exam revealed a correlation ranging from 49 % to 103 % between estimated (thermal map) and resulting (24 h CEMR) necrosis (Figure 5). At the same time only median 39 % (range 16 % - 78 %) agreement was calculated for T1 signal loss and consecutive necrosis in the matched control group of T1 magnitude thermal monitoring. Median underestimation of the size of necrosis in the PRFS group was as much as 21 % (range 1 % - 52 %). Overestimation in two cases 2.2 % on average. The most accurate prediction of consecutive necrosis in the T1 magnitude group was 78 % of the size with a median underestimation of 61 % (range 22 % - 84 %), representing the overall predictive error in this group (Figure 6). In comparison the overall predictive error in the PRFS group remains 21 % (range 1 % - 52 %), taking into account the two cases of slight overestimation. The difference in estimating tissue necrosis between the two thermometric modalities was significant (p = 0.004), whereas the extent of therapy-related necrosis, as evident in the first-day control exam, in the two groups was not significantly different (p > 0.28). At the same time the Spearman's rank test verified a positive correlation between zones of lethal thermal impact (thermal map) and ensued necrosis (24 h CEMR) both in the PRFS group (correlation coefficient 0.69) and the T1 magnitude group (correlation coefficient 0.62). Correlations were statistically significant (p < 0.0004).

## Discussion

### Comparative matched-pair analysis

PRFS-based MR thermometry has become the preferred technique to facilitate online monitoring of thermal ablation. The GRE sequence used for thermometry in this study has been described and validated before [26]. To the knowledge of the authors this is the first report on the employment in a clinical routine set-up of laser ablation in human livers. Now the focus is on performance comparison with an older thermometry technique based on the operability of the online interface in the model. The results show the PRFS method´s superiority over the competing T1 magnitude method. The earlier T1 method holds an almost three-fold predictive error of median 61 % compared with the newer PRFS approach. Favorising the PRFS method as a result of this study’s comparative analysis confirms findings from several preclinical trials [4,6,23,35,36]. Actual findings are supported by coexisting fair statistical correlations between predictive medium (thermal map) and control (24 h CEMR) in the compared models. Terraz et al., who investigated a similar sequence design monitoring RFA, where not able to show such correlation in their data pool when comparing lethal dose area and necrosis as shown in the follow-up CEMR [37]. Obviously qualitative and quantitative assessment of a method´s performance, in the given design, requires a preferably linear relationship of the two variables investigated. It has to be taken into account that, investigating both a method´s intrinsic accuracy and that of the virtual online visualization interface, a two-fold potential mismatch may play a role. Sensitive elements of the implementation used for this study are size and position of the characteristic ROI displaying the thermal map in the PRFS model. As shown with single cases in this study the fix ROI, optionally 10 or 15 cm^2^ with a given FOV, may cut off areas of hypothetically lethal thermal impact, resulting in “thermometrically blind” areas (Figure 4). This automatically accounted for an underestimation of tissue necrosis in these cases, but could not be quantified in the chosen study design – the resulting bias was tolerated as the positive correlation of lethal thermal zone and necrosis stayed statistically significant. Also the representative peak temperature image chosen for evaluation may have been slightly out of plane without any relevant internal control. Breathing position in the triggered thermometric GRE and any other sequence used for planning must be carefully leveled. Together, sizing and positioning of the ROI momentarily account for a human factor influencing the PRFS method´s accuracy. Both phenomena may have partly caused an increase of the predictive error and be ruled out in future multiplane or 3D work-ups of the software and through free online relocatability of the ROI. 

### Accuracy of the PRFS thermometry display

With a predictive error of 21 % between a monitoring tool and the resulting necrosis the predictive value of the computed human-software interface, present to the interventional radiologist at site, does not fully satisfy expectations revealed by the postprocessing evaluation of the same method [26]. There semiautomated postprocessing of whole phase image and consecutive necrosis in the PRFS group showed an 87 % conformity at temperatures above 52 °C. False-negative underestimation of necrosis and false-positive overestimation of necrosis were 13 % on average each. Three major differences in the analytic work-up may account for the different outcome: Firstly, postprocessing of the phase difference image was not limited to a fix ROI, where borders potentially cut off areas of lethal impact. Secondly, temperature values for all voxels had been corrected based on the Pennes bioheat equation leveling image noise as well as intra- and interscan motion artifacts and therefore diminishing temperature standard deviation by a factor of 1.9 as compared with that of primarily measured peak temperature values. Thirdly, the temperature threshold was set to 52 °C, three degrees cooler than in the present work-up, based on employment of the Arrhenius integral to calculate a lethal dose. Earlier Rempp et al. reported comparably good results applying the GRE sequence used in this study with RFA and a temperature threshold of 60 °C [25]. With respect to both studies the temperature threshold had been set to 55 °C in the actual study work-up. Taking into account results of Rempp and Kickhefel, whose work is limited to semi-quantitative postprocessing assessment of whole image data, it can be assumed, from findings of this study, that the accuracy of the model’s online presenting tool by now is slightly inferior to the method itself; even though the influence of different study designs is plausible.

Accuracy mismatch between methodical and presentational tools are not a singular phenomenon. Terraz et al. used temperature maps based on a semi-quantitative method. Calculating a lethal dose according to Sapatero and Dewey’s computed a homogeneous mask that was laid over the GRE magnitude image qualitatively determinating “in” or “out” [37]. Calculated temperature thresholds are not directly clarified. The authors report the need of reablation in 3 of 7 liver tumors based on evaluation of the mapping interface. Quantifying the area of interest for the online tool failed to show a correlation with later necrosis. Garcia-Medina et al. report on a both quantitative and qualitative interface synchronously decoding an Arrhenius algorithm to monitor laser ablation in living pigs [38]. But the article remains somewhat diffuse on the determinants and accuracy of the method.

At the same time it has to be taken into account that there is very few relevant reporting of comparable in vivo data for monitoring laser or even thermal therapy in moving organs and in a clinical set-up. Implemented thermometric approaches, until now, never enabled immediate evaluation of treatment outcome, which is confirmed by the results of this comparative study showing inadequately low predictive values for the alternative T1 magnitude method. Comparative studies at this stage represent the steep part of an asymptotic approximation in achieving a sufficient monitoring and controlling tool for thermal tumor ablation. Adjusting the prototype version of the PRFS model presented is expected to further enhance its performance.

The authors believe the present study to confirm the feasibility of thermal ablation of small liver tumors being performed entirely under MR guidance and utilizing PRFS thermometry. As mentioned before, convenient conditions have also been reported for the use of RFA and adequate MR filter systems in human livers [24,25]. In contrast to RFA, which interferes with the magnetic field, LA does not depend on the use of MR filters for real-time or near real-time in-bore imaging. Also the glass fiber that is used for LA, as shown in this study, does not induce any metal artifact otherwise originating from the RFA probe. Lepetit-Coiffé et al. in their study report an RFA electrode artifact ranging between 14 and 17 mm while treating tumors with a median diameter of 18 mm [24]. These factors may be advantageous using PRFS temperature mapping with advanced spatial resolution to monitor laser-induced thermal ablation, even though modality comparison was not part of the actual study.

The primary aim remains to be success evaluation at the time of ablation [11,27]. Parameters should be device-independent, e.g. not a stationary feature of the instrument that is used.

### Limitations

A limitation of this study was the lack of histological work-up of treated liver tissue, which related to the non-surgical treatment approach for included inoperable patients. Groups were not randomized. With respect to feasibility aspects 24 h control imaging was chosen as primary endpoint of the study. Further follow-up of the patients will be investigated even though it is not discriminating between the two methods being comparatively analyzed. Ablation cut-off points were not primarily chosen through interpretation of real-time thermal imaging but in correlation with a standard treatment regimen. At the same time, no adverse event occurred that would have influenced thermal mapping or ablation duration. Volume measurements and 3D analysis could not be performed due to single-plane thermal map acquisition. For the same reason anatomic correlation, e.g. with vasculature, was not part of the study. With respect to guidelines of standardization [11] the approach of data acquisition is legitimate to evaluate online visualization being the tool used by the interventional radiologist during the procedure.

## Supporting Information

Table S1 **Matched pairs of PRFS-guided (A) vs. T1-magnitude-guided (B) ablative procedures.**
(DOCX)

## References

[1] Chen X, Barkauskas KJ, Nour SG, Duerk JL, Abdul-Karim FW et al. (2007) Magnetic resonance imaging and model prediction for thermal ablation of tissue. J Magn Reson Imaging 26: 123-132. doi:10.1002/jmri.20956. PubMed: 17659563. 17659563

[2] Clasen S, Pereira PL (2008) Magnetic resonance guidance for radiofrequency ablation of liver tumors. J Magn Reson Imaging 27: 421-433. doi:10.1002/jmri.21264. PubMed: 18219677. 18219677

[3] de Senneville BD, Mougenot C, Quesson B, Dragonu I, Grenier N et al. (2007) MR thermometry for monitoring tumor ablation. Eur Radiol 17: 2401-2410. doi:10.1007/s00330-007-0646-6. PubMed: 17701184. 17701184

[4] Germain D, Chevallier P, Laurent A, Saint-Jalmes H (2001) MR monitoring of tumour thermal therapy. Magma 13: 47-59. doi:10.1007/BF02668650. PubMed: 11410396. 11410396

[5] Peters RD, Chan E, Trachtenberg J, Jothy S, Kapusta L et al. (2000) Magnetic resonance thermometry for predicting thermal damage: an application of interstitial laser coagulation in an in vivo canine prostate model. Magn Reson Med 44: 873-883. doi:10.1002/1522-2594(200012)44:6. PubMed: 11108624. 11108624

[6] Rieke V, Butts Pauly K (2008) MR thermometry. J Magn Reson Imaging 27: 376-390. doi:10.1002/jmri.21265. PubMed: 18219673. 18219673 PMC2780364

[7] Kim JH, Hahn EW (1979) Clinical and biological studies of localized hyperthermia. Cancer Res 39: 2258-2261. PubMed: 445426. 445426

[8] Thomsen S (1991) Pathologic analysis of photothermal and photomechanical effects of laser-tissue interactions. Photochem Photobiol 53: 825-835. PubMed: 1886941. 1886941 10.1111/j.1751-1097.1991.tb09897.x

[9] Cheng HL, Plewes DB (2002) Tissue thermal conductivity by magnetic resonance thermometry and focused ultrasound heating. J Magn Reson Imaging 16: 598-609. doi:10.1002/jmri.10199. PubMed: 12412038. 12412038

[10] Han B, Hanson WL, Bensalah K, Tuncel A, Stern JM et al. (2009) Development of quantum dot-mediated fluorescence thermometry for thermal therapies. Ann Biomed Eng 37: 1230-1239. doi:10.1007/s10439-009-9681-6. PubMed: 19322658. 19322658

[11] Goldberg SN, Grassi CJ, Cardella JF, Charboneau JW, Dodd GD 3rd et al. (2009) Image-guided tumor ablation: standardization of terminology and reporting criteria. J Vasc Interv Radiol 20: 377-390. doi:10.1016/j.jvir.2009.04.011. PubMed: 195600261594704015845798. 15947040

[12] Chen J, Daniel BL, Diederich CJ, Bouley DM, van den Bosch MA et al. (2008) Monitoring prostate thermal therapy with diffusion-weighted MRI. Magn Reson Med 59: 1365-1372. doi:10.1002/mrm.21589. PubMed: 18506801. 18506801

[13] Eyrich GK, Bruder E, Hilfiker P, Dubno B, Quick HH et al. (2000) Temperature mapping of magnetic resonance-guided laser interstitial thermal therapy (LITT) in lymphangiomas of the head and neck. Lasers Surg Med 26: 467-476. doi:10.1002/1096-9101(2000)26:5. PubMed: 10861702. 10861702

[14] Grissom WA, Kerr AB, Holbrook AB, Pauly JM, Butts-Pauly K (2009) Maximum linear-phase spectral-spatial radiofrequency pulses for fat-suppressed proton resonance frequency-shift MR Thermometry. Magn Reson Med 62: 1242-1250. doi:10.1002/mrm.22118. PubMed: 19780177. 19780177 PMC2795148

[15] Holbrook AB, Santos JM, Kaye E, Rieke V, Pauly KB (2010) Real-time MR thermometry for monitoring HIFU ablations of the liver. Magn Reson Med 63: 365-373. doi:10.1002/mrm.22206. PubMed: 19950255. 19950255 PMC3212435

[16] Li C, Pan X, Ying K, Zhang Q, An J et al. (2009) An internal reference model-based PRF temperature mapping method with Cramer-Rao lower bound noise performance analysis. Magn Reson Med 62: 1251-1260. doi:10.1002/mrm.22121. PubMed: 19780176. 19780176

[17] McDannold N, Tempany C, Jolesz F, Hynynen K (2008) Evaluation of referenceless thermometry in MRI-guided focused ultrasound surgery of uterine fibroids. J Magn Reson Imaging 28: 1026-1032. doi:10.1002/jmri.21506. PubMed: 18821603. 18821603 PMC2574694

[18] Nour SG, Lewin JS (2005) Radiofrequency thermal ablation: the role of MR imaging in guiding and monitoring tumor therapy. Magn Reson Imaging Clin N Am 13: 561-581. doi:10.1016/j.mric.2005.04.007. PubMed: 16084420. 16084420

[19] Rieke V, Vigen KK, Sommer G, Daniel BL, Pauly JM et al. (2004) Referenceless PRF shift thermometry. Magn Reson Med 51: 1223-1231. doi:10.1002/mrm.20090. PubMed: 15170843. 15170843

[20] Scheffler K (2004) Fast frequency mapping with balanced SSFP: theory and application to proton-resonance frequency shift thermometry. Magn Reson Med 51: 1205-1211. doi:10.1002/mrm.20081. PubMed: 15170841. 15170841

[21] Vogel MW, Pattynama PM, Lethimonnier FL, Le Roux P (2003) Use of fast spin echo for phase shift magnetic resonance thermometry. J Magn Reson Imaging 18: 507-512. doi:10.1002/jmri.10393. PubMed: 14508789. 14508789

[22] Streitparth F, Hartwig T, Walter T, De Bucourt M, Putzier M et al. (2013) MR guidance and thermometry of percutaneous laser disc decompression in open MRI: an initial clinical investigation. Eur Radiol, 23: 2739–46. doi:10.1007/s00330-013-2872-4. PubMed: 23657288. 23657288

[23] Cernicanu A, Lepetit-Coiffe M, Roland J, Becker CD, Terraz S (2008) Validation of fast MR thermometry at 1.5 T with gradient-echo echo planar imaging sequences: phantom and clinical feasibility studies. NMR Biomed 21: 849-858. doi:10.1002/nbm.1267. PubMed: 18574794. 18574794

[24] Lepetit-Coiffé M, Laumonier H, Seror O, Quesson B, Sesay MB et al. (2010) Real-time monitoring of radiofrequency ablation of liver tumors using thermal-dose calculation by MR temperature imaging: initial results in nine patients, including follow-up. Eur Radiol 20: 193-201. doi:10.1007/s00330-009-1532-1. PubMed: 19657650. 19657650

[25] Rempp H, Hoffmann R, Roland J, Buck A, Kickhefel A et al. (2012) Threshold-based prediction of the coagulation zone in sequential temperature mapping in MR-guided radiofrequency ablation of liver tumours. Eur Radiol 22: 1091-1100. doi:10.1007/s00330-011-2335-8. PubMed: 22105843. 22105843

[26] Kickhefel A, Rosenberg C, Weiss CR, Rempp H, Roland J et al. (2011) Clinical evaluation of MR temperature monitoring of laser-induced thermotherapy in human liver using the proton-resonance-frequency method and predictive models of cell death. J Magn Reson Imaging 33: 704-712. doi:10.1002/jmri.22499. PubMed: 21563256. 21563256

[27] Schmiegel W, Pox C, Reinacher-Schick A, Adler G, Arnold D et al. (2010) S3 guidelines for colorectal carcinoma: results of an evidence-based consensus conference on February 6/7, 2004 and June 8/9, 2007 Z Gastroenterol 48:65-136 10.1055/s-0028-1109936 20072998

[28] Puls R, Stroszczynski C, Rosenberg C, Kuehn JP, Hegenscheid K et al. (2007) Three-dimensional gradient-echo imaging for percutaneous MR-guided laser therapy of liver metastasis. J Magn Reson Imaging 25: 1174-1178. doi:10.1002/jmri.20936. PubMed: 17520737. 17520737

[29] Rosenberg C, Puls R, Hegenscheid K, Kuehn J, Bollman T et al. (2009) Laser ablation of metastatic lesions of the lung: long-term outcome. AJR 192: 785-792. doi:10.2214/AJR.08.1425. PubMed: 19234278. 19234278

[30] Parker DL (1984) Applications of NMR imaging in hyperthermia: an evaluation of the potential for localized tissue heating and noninvasive temperature monitoring. IEEE Trans Biomed Eng 31: 161-167. PubMed: 6724602. 6724602 10.1109/TBME.1984.325382

[31] Puls R, Langner S, Rosenberg C, Hegenscheid K, Kuehn JP et al. (2009) Laser ablation of liver metastases from colorectal cancer with MR thermometry: 5-year survival. J Vasc Interv Radiol 20: 225-234. doi:10.1016/j.jvir.2008.10.018. PubMed: 19109037. 19109037

[32] Kuhn JP, Puls R, Wallaschowski H, Heidecke CD, Rosenberg C et al. (2008) Characteristics of necrosis after laser-induced thermotherapy in contrast-enhanced MRI and implications for treatment success. Fortschr Roentgenstr 180: 816-820. doi:10.1055/s-2008-1027478. 18671194

[33] Dietrich O, Raya JG, Reeder SB, Reiser MF, Schoenberg SO (2007) Measurement of signal-to-noise ratios in MR images: influence of multichannel coils, parallel imaging, and reconstruction filters. J Magn Reson Imaging 26: 375-385. doi:10.1002/jmri.20969. PubMed: 17622966. 17622966

[34] Firbank MJ, Harrison RM, Williams ED, Coulthard A (2000) Quality assurance for MRI: practical experience. Br J Radiol 73: 376-383. PubMed: 10844863. 10844863 10.1259/bjr.73.868.10844863

[35] Kickhefel A, Roland J, Weiss C, Schick F (2010) Accuracy of real-time MR temperature mapping in the brain: a comparison of fast sequences. Phys Med 26: 192-201. doi:10.1016/j.ejmp.2009.11.006. PubMed: 20096617. 20096617

[36] Meister D, Hubner F, Mack M, Vogl TJ (2007) MR thermometry for laser-induced thermotherapy at 1.5 Tesla. Fortschr Roentgenstr 179: 497-505. doi:10.1055/s-2007-962979. 17436184

[37] Terraz S, Cernicanu A, Lepetit-Coiffé M, Viallon M, Salomir R et al. (2010) Radiofrequency ablation of small liver malignancies under magnetic resonance guidance: progress in targeting and preliminary observations with temperature monitoring. Eur Radiol 20: 886-897. doi:10.1007/s00330-009-1611-3. PubMed: 19760231. 19760231

[38] Garcia-Medina O, Gorny K, McNichols R, Friese J, Misra S et al. (2011) In vivo evaluation of a MR-guided 980nm laser interstitial thermal therapy system for ablations in porcine liver. Lasers Surg Med 43: 298-305. doi:10.1002/lsm.21044. PubMed: 21500224. 21500224

