# Supplementary material for: PRFS-Based MR Thermometry Versus an Alternative T1 Magnitude Method – Comparative Performance Predicting Thermally Induced Necrosis in Hepatic Tumor Ablation
Source: PLoS One. 2013 Oct 24;8(10):e78559. doi: 10.1371/journal.pone.0078559 (PMC3813475; doi:10.1371/journal.pone.0078559)
Supplement: Table S1 — Matched pairs of PRFS-guided (A) vs. T1-magnitude-guided (B) ablative procedures. (DOCX) [file pone.0078559.s001.docx]

Table S1. Matched pairs of PRFS-guided (A) vs. T1-magnitude-guided (B) ablative procedures.

| **Pairs** | **Patient** | **Age (yrs)** | **Target (cm^2^)** | **Primary tumor type** | **Localization (segment)** | **Applicators (n)** | **Applied energy (kJ)** |
| --- | --- | --- | --- | --- | --- | --- | --- |
|  |  |  |  |  |  |  |  |
| 1 | A | 80 | 4.2x3.0 | CC | 7 | 2 | 32 |
|  | B | 83 | 4.5x4.4 | CC | 7 | 3 | 21.2 |
| 2 | A | 61 | 0.5x0.5 | CC | 7 | 1 | 18.4 |
|  | B | 42 | 0.7x0.5 | BC | 7 | 1 | 22.2 |
| 3 | A | 69 | 1.2x1.2 | HCC | 5 | 2 | 44.1 |
|  | B | 70 | 1.0x1.0 | CC | 7 8 | 2 | 52.3 |
| 4 | A | 66 | 3.3x2.5 | CC | 8 | 2 | 37.8 |
|  | B | 55 | 3.1x3.0 | CC | 8 | 2 | 40 |
| 5 | A | 72 | 2.0x1.9 | CC | 4 | 2 | 41.9 |
|  | B | 68 | 2.5x1.8 | CC | 4 8 | 3 | 28.8 |
| 6 | A | 66 | 2.8x2.5 | CC | 6 | 3 | 65.2 |
|  | B | 66 | 2.5x.2.7 | CC | 7 | 2 | 33 |
| 7 | A | 69 | 1.2x1.2 | HCC | 5 | 2 | 24.7 |
|  | B | 76 | 0.9x0.9 | HCC | 7 | 2 | 34.7 |
| 8 | A | 56 | 1.0x1.0 | CC | 5 | 2 | 37.7 |
|  | B | 62 | 1.5x1.3 | CC | 8 5 | 2 | 15.5 |
| 9 | A | 66 | 1.8x1.5 | CC | 6 | 2 | 24.1 |
|  | B | 68 | 1.6x1.5 | HCC | 7 | 2 | 24 |
| 10 | A | 61 | 1.3x1.0 | CC | 6 | 2 | 37.8 |
|  | B | 51 | 1.2x1.0 | CC | 7 | 2 | 25.7 |
| 11 | A | 67 | 1.9x1.8 | CC | 8 | 2 | 27.9 |
|  | B | 82 | 1.7x1.5 | CC | 8 | 1 | 25.1 |
| 12 | A | 67 | 2.4x2.2 | EC | 6 | 2 | 32 |
|  | B | 55 | 2.0x1.8 | BC | 6 7 | 3 | 44.6 |
| 13 | A | 67 | 2.8x2.5 | EC | 6 | 3 | 48.1 |
|  | B | 82 | 2.8x2.4 | CC | 6 7 | 2 | 23 |
| 14 | A | 67 | 2.5x2.0 | EC | 6 | 2 | 32.4 |
|  | B | 62 | 2.5x2.2 | BC | 6 | 2 | 38 |
| 15 | A | 61 | 1.6x1.5 | CC | 6 | 2 | 34.8 |
|  | B | 50 | 2.0x1.8 | NPC | 6 | 2 | 18 |
| 16 | A | 52 | 1.4x1.1 | CC | 5 6 | 1 | 12.2 |
|  | B | 64 | 1.0x1.0 | CC | 7 | 2 | 20.7 |
| 17 | A | 61 | 2.1x2.0 | CC | 2 | 2 | 32 |
|  | B | 37 | 3.1x2.9 | CC | 3 | 3 | 60.1 |
| 18 | A | 68 | 1.8x1.6 | CC | 2 | 2 | 32 |
|  | B | 62 | 2.3x2.0 | CC | 3 | 2 | 36 |
| 19 | A | 72 | 4.4x4.2 | CC | 8 | 4 | 62 |
|  | B | 66 | 3.9x3.8 | CC | 7 | 4 | 65.6 |
| 20 | A | 68 | 0.6x0.6 | CC | 4 | 1 | 16 |
|  | B | 71 | 1.3x1.0 | CC | 4 | 2 | 30 |
| 21 | A | 69 | 2.0x1.5 | CC | 4 | 2 | 32 |
|  | B | 70 | 2.5x2.3 | PC | 8 | 3 | 63.8 |
| 22 | A | 52 | 1.5x1.5 | PC | 3 | 1 | 18 |
|  | B | 62 | 2.9x2.3 | BC | 2 3 | 2 | 25.1 |
| 23 | A | 60 | 1.7x1.5 | CC | 2 | 2 | 29.5 |
|  | B | 42 | 1.2x1.2 | BC | 6 | 1 | 20.8 |
| 24 | A | 61 | 1.7x1.5 | CC | 2 | 1 | 16 |
|  | B | 76 | 1.2x1.2 | HCC | 2 | 1 | 16 |
| 25 | A | 72 | 4.0x3.8 | CC | 8 | 4 | 64.2 |
|  | B | 51 | 4.4x4.0 | CC | 8 | 2 | 32 |
| 26 | A | 72 | 2.5x2.1 | CC | 5 | 2 | 31.9 |
|  | B | 80 | 2.5x2.5 | CC | 5 6 | 2 | 25.1 |
| 27 | A | 58 | 2.5x2.1 | CC | 2 | 3 | 44.3 |
|  | B | 80 | 2.4x2.2 | CC | 7 | 2 | 24.6 |
| 28 | A | 70 | 3.8x3.5 | MM | 6 7 8 | 3 | 48 |
|  | B | 66 | 3.7x3.1 | PC | 8 | 2 | 32 |
| 29 | A | 58 | 1.2x1.0 | CC | 4 | 2 | 32.1 |
|  | B | 61 | 1.9x1.4 | CC | 8 | 2 | 30.4 |
| 30 | A | 57 | 3.2x3.1 | BC | 6 | 3 | 36.3 |
|  | B | 60 | 3.7x3.1 | NC | 8 | 4 | 50.9 |
| 31 | A | 70 | 3.4x3.0 | CCC | 6 7 | 4 | 64.3 |
|  | B | 71 | 3.2x3.1 | CCC | 6 | 1 | 18.8 |
| 32 | A | 67 | 5.6x5.0 | GC | 8 | 4 | 64.2 |
|  | B | 62 | 6.0x3.7 | BC | 2 3 | 3 | 69 |
| 33 | A | 69 | 1.9x1.7 | CC | 4 8 | 2 | 30.6 |
|  | B | 65 | 1.2x0.8 | CC | 8 | 2 | 35 |
| 34 | A | 53 | 2.2x1.4 | CC | 6 | 2 | 32 |
|  | B | 71 | 1.4x1.2 | CC | 7 | 2 | 48.2 |

CC=Colorectal carcinoma, BC=Breast carcinoma, HCC=Hepatocellular carcinoma, EC=Endometrium carcinoma,

NPC=Nasopharyngeal carcinoma, PC= Pancreatic carcinoma, NC=Neuroendocrine carcinoma,

MM=Malignant melanoma, CCC=Cholangiocellular carcinoma
